# A Gene Expression Profile Test for the Differential Diagnosis of Ovarian Versus Endometrial Cancers

**DOI:** 10.18632/oncotarget.450

**Published:** 2012-02-23

**Authors:** Anita Lal, Rebecca Panos, Mira Marjanovic, Michael Walker, Eloisa Fuentes, Daniel S Kapp, W. David Henner, Ljubomir J. Buturovic, Meredith Halks-Miller

**Affiliations:** ^1^ Pathwork Diagnostics, Redwood City, CA; ^2^ Department of Radiation Oncology, Stanford University, Stanford, CA

**Keywords:** gene expression, tissue of origin, endometrial cancer, ovarian cancer, diagnostic test

## Abstract

We have developed a gene expression profile test (Pathwork Tissue of Origin Endometrial Test) that distinguishes primary epithelial ovarian and endometrial cancers in formalin-fixed, paraffin-embedded (FFPE) specimens using a 316–gene classification model. The test was validated in a blinded study using a pre-specified algorithm and microarray files for 75 metastatic, poorly differentiated or undifferentiated specimens with a known ovarian or endometrial cancer diagnosis. Measures of test performance include a 94.7% overall agreement with the known diagnosis, an area under the ROC curve (AUC) of 0.997 and a diagnostic odds ratio (DOR) of 406. Ovarian cancers (n=30) gave an agreement of 96.7% with the known diagnosis while endometrial cancers (n=45) gave an agreement of 93.3%. In a precision study, concordance in test results was 100%. Reproducibility in test results between three laboratories was 94.3%. The Tissue of Origin Endometrial Test can aid in resolving important differential diagnostic questions in gynecologic oncology.

## INTRODUCTION

Accurate diagnosis of ovarian and endometrial cancer impacts not only prognosis and clinical management of these patients but also anticancer drug and radiation therapy choices as well as entry opportunities for clinical trials. Treatment regimens for endometrial cancer patients often include radiation therapy and possibly hormonal therapy. Ovarian cancer patients are typically managed differently from endometrial cancer patients and undergo meticulous surgery including aspiration of ascites or peritoneal lavage, attempted optimal tumor debulking and, if no gross extraovarian disease is visualized, random peritoneal biopsies [1,2]. Often, the differential diagnosis of ovarian versus endometrial cancers is a challenging problem in diagnostic gynecologic pathology. Ascertaining the primary gynecologic site of a metastatic carcinoma is sometimes difficult because specific histologic subtypes of ovarian and endometrial cancers appear similar on gross and microscopic examination. For example, ovarian endometrioid carcinomas, which constitute up to 13% of ovarian cancers, are histologically similar to endometrial endometrioid carcinomas [3-5]. Similarly, endometrial serous adenocarcinomas are commonly confused with ovarian serous adenocarcinomas [6].

Endometrial cancers commonly metastasize to the ovary and often mimic an ovarian primary [7,8]. In cases of disseminated cancers that involve both the ovary and the endometrium, it is often impossible to establish whether the cancer represents metastatic spread from a uterine primary, metastatic spread from an ovarian primary or whether it represents synchronous ovarian and endometrial primaries [9]. Poorly differentiated and undifferentiated carcinomas, in particular, can be difficult to diagnose accurately using morphological criteria [10]. There is a clear need for ancillary techniques beyond microscopic evaluations of routine hematoxylin and eosin (H&E) stained sections that would enable pathologists to make an accurate ovarian or endometrial cancer diagnosis.

Immunohistochemistry (IHC) is the most commonly used ancillary technique in gynecologic pathology. Immunohistochemical biomarkers are typically not specific for a single tumor type necessitating the use of a panel of antibodies [7,11,12]. In the case of ovarian and endometrial cancers, immunohistochemical biomarkers have overlapping immunoreactivities with specific histologic subtypes of both ovarian and endometrial cancers. For example, p16 immunoreactivity is observed in both ovarian and endometrial serous adenocarcinomas and is largely absent from both ovarian and endometrial endometrioid adenocarcinomas [13]. Yet other biomarkers, such as WT1, are specific to only a single histologic subtype within either ovarian or endometrial cancers. Ovarian serous adenocarcinomas exhibit positive immunostaining with WT1 while endometrial serous carcinomas and both ovarian and endometrial endometrioid adenocarcinomas show negative immunostaining with WT1 [6]. A large proportion of ovarian cancers, with the exception of ovarian mucinous adenocarcinomas, are positive for estrogen and progesterone receptors [13]. However, a considerable proportion of endometrial cancers are also positive for these receptors precluding the utility of these common biomarkers to distinguish ovarian from endometrial cancers [14]. Given the morphologic heterogeneity of ovarian and endometrial cancers and the complex pattern of expression of immunohistochemical biomarkers in these cancers, additional diagnostic approaches are required for their accurate classification.

In addition to the challenges that pathologists face in using IHC to distinguish between ovarian and endometrial cancer, validation of antibodies used in IHC is expensive, time-consuming and performed inconsistently. Interpretation and reporting of IHC results are also subjective and user-dependent. Molecular diagnostic tests that use gene expression profiling with microarrays to classify cancers according to their primary sites are now a feasible tool for cancer diagnosis [15-18]. Advances in gene annotation and array design along with the use of standardized protocols and array platforms across laboratories have made microarray-based gene expression profiling extremely reproducible [16,19-21]. These assays have the advantage of measuring the expression of a multitude of biomarkers simultaneously [22]. Additionally, the use of RNA from formalin-fixed paraffin embedded (FFPE) tissue in microarray-based diagnostics has become more common, considerably expanding the utility of these diagnostic tests [16,23-25].

We have developed a microarray-based diagnostic test, the Pathwork Tissue of Origin Endometrial Test (Pathwork Diagnostics, Redwood City, CA), that can be used for the differential diagnosis of ovarian versus endometrial cancers. We present data from a blinded, multicenter clinical validation study showing that the Tissue of Origin Endometrial Test has a high accuracy of 94.7% in classifying ovarian and endometrial cancers. We also show that inter-laboratory reproducibility of test results was 94.3%. The Tissue of Origin Endometrial Test is a novel molecular diagnostic test that is expected to be a valuable aid to physicians treating patients of gynecologic cancers.

## RESULTS

### The Tissue of Origin Endometrial Test

The Tissue of Origin Endometrial Test measures the expression of 375 probesets (316 independent genes) that serve as markers during classification of ovarian and endometrial cancers. Sensitivity, specificity and AUC values for the training data were calculated using cross-validation for different numbers of classification probesets (Figure 2). The performance curves converge to their asymptotic values at just under 400 probesets. We chose the simplest model in our judgment consisting of 375 probesets and providing maximum achievable predictive performance on this platform. Even though the classification markers are empirically selected by machine learning, they included several genes that were previously known to have a function in the biology of ovarian and endometrial cancers (Table 2). Homeobox A10 (HOXA10) and Homeobox A11 (HOXA11) have previously been shown to be overexpressed in ovarian cancers, and play a role in stimulating tumor growth and determining the histologic identity of epithelial ovarian cancers [26-28]. Specific homologs of the Kallikrien peptidases have been evaluated as serum diagnostic markers for the early detection of both ovarian and endometrial cancers [29-32]. WT1 is an immunohistochemical biomarker used for distinguishing ovarian versus endometrial serous adenocarcinomas [6].

**Figure 1:**
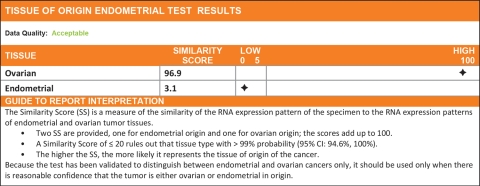
A sample Tissue of Origin Endometrial Test report The report presents 2 similarity scores, one for ovarian and one for endometrial cancers. The two similarity scores add up to 100. The tissue type with the higher similarity score is the more likely tissue of origin. In the sample shown, ovarian cancer with a similarity score of 96.9 is the more likely tissue of origin.

**Figure 2:**
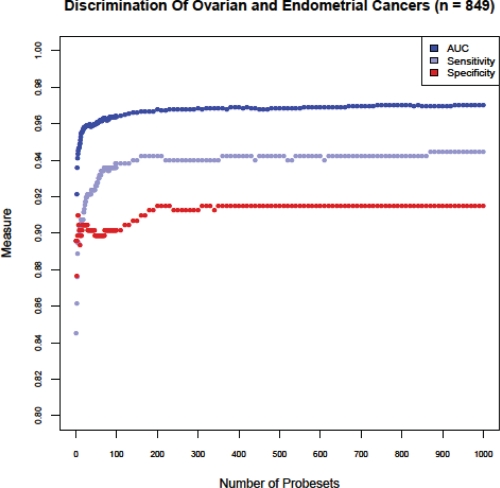
Determination of the optimal number of classification probesets for the Tissue of Origin Endometrial Test Sensitivity, Specificity and AUC were analyzed using cross-validation of training data for different numbers of classification probesets. A model with 375 probesets was chosen because performance curves had reached asymptotic values and maximal predictive performance was achieved.

**Table 1:** Morphologies on the Tissue of Origin Endometrial Test Panel

Tissue Type	Included Histologic Subtypes*	Excluded Histologic Subtypes*
**Endometrial**	Adenocarcinoma, NOS	
	Clear cell adenocarcinoma	Endometrial stromal sarcoma
	Endometrioid adenocarcinoma	Leiomyosarcoma
	Serous adenocarcinoma	
	Mixed cell adenocarcinoma	
	Malignant müllerian mixed tumor	
**Ovarian**	Adenocarcinoma, NOS	
	Clear cell adenocarcinoma	Primitive germ cell tumors
	Endometrioid adenocarcinoma	Sex cord-stromal tumors
	Mixed epithelial tumor	Malignant Brenner tumor
	Mucinous adenocarcinoma	Malignant müllerian mixed tumor
	Serous adenocarcinoma	
	Serous surface papillary adenocarcinoma	
	Undifferentiated carcinoma	

* Histologic subtypes are listed using WHO nomenclature

**Table 2:** Top 10 Biomarkers in the Tissue of Origin Endometrial Test Classification Algorithm

Marker Rank	Probeset ID	Gene Symbol	Gene Name	Cellular Function
**1**	213147_at	HOXA10*	Homeobox A10	Transcription Factor
**2**	213707_s_at	DLX5	Distal-less homeobox 5	Transcription Factor
**3**	213823_at	HOXA11*	Homeobox A11	Transcription Factor
**4**	204733_at	KLK6*	Kallikrein-related peptidase 6	Serine protease
**5**	206125_s_at	KLK8*	Kallikrein-related peptidase 8	Serine protease
**6**	207076_s_at	ASS1	Argininosuccinate synthase 1	Arginine Biosynthesis
**7**	205778_at	KLK7*	Kallikrein-related peptidase 7	Serine protease
**8**	32625_at	NPR1	Natriuretic peptide receptor A/ guanylate cyclase A	
**9**	214844_s_at	DOK5	Docking protein 5	Adaptor protein of MAP Kinase Pathway
**10**	206067_s_at	WT1*^$^	Wilms tumor 1	Transcription Factor

* Previously known to have a functional role in ovarian and/or endometrial cancers

$ Biomarker known to distinguish ovarian and endometrial serous adenocarcinomas

### Processing of Clinical Validation Specimens

Tissue sections from 82 specimens were processed for total RNA. Thirty nanograms of total RNA is a required quality metrics to perform the Tissue of Origin Endometrial Test. Eighty one specimens that yielded at least 30 ng of total RNA at a concentration of ≥ 9.5 ng/μl and had an A_260_/A_280_ ratio of ≥ 1.0 were processed further. Of these, 80 specimens yielded at least 2.5 μg of biotinylated cDNA, which is a required cDNA quality metrics, and were hybridized to Pathchip microarrays. Of the 80 specimens, 79 specimens passed pre-specified microarray data quality criteria. In all, 96.3% (79/82) of FFPE specimens processed passed all quality criteria for the Tissue of Origin Endometrial Test. Four additional specimens were subsequently removed because the specimen vendor did not follow standard quality control procedures and these specimens did not meet specimen entry criteria of a known ovarian or endometrial clinical diagnosis. A total of 75 specimens were used in data analysis.

### Performance of the Tissue of Origin Endometrial Test

The clinical validation study followed a Bayesian adaptive design. Testing was terminated at Look1 because the acceptance criteria of mean PPA and lower bound of the 95% credible interval for both ovarian and endometrial cancers exceeded pre-specified threshold values at this interim analysis (Supplementary Table 2). The positive percent agreement (PPA) and percent non-agreement with 95% confidence intervals for the Tissue of Origin Endometrial Test are summarized in Table 3. The overall agreement of the Tissue of Origin Endometrial Test results with the known clinical diagnosis was 94.7% (95% CI, 86.9% to 98.5%). Ovarian cancers had a higher PPA of 96.7% compared to endometrial cancers that had a PPA of 93.3% (Fisher's exact test, p=0.646; Table 3).

**Table 3:** Accuracy of the Tissue of Origin Endometrial Test

Known Clinical Diagnosis	Positive Percent Agreement Percent (ratio) [95% Confidence Interval]	Percent Non-Agreement Percent (ratio) [95% Confidence Interval]
**Endometrial**	93.3 (42/45) [81.7-98.6]	6.7 (3/45) [1.4-18.3]
**Ovarian**	96.7 (29/30) [82.8-99.9]	3.3 (1/30) [0.1-17.2]
**Overall**	94.7 (71/75) [86.9-98.5]	5.3 (4/75) [1.5-13.1]

**Figure 3:**
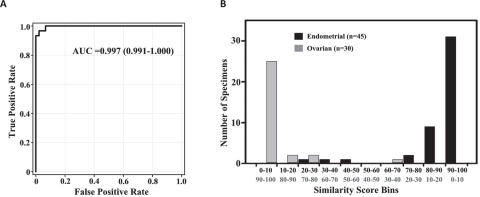
Performance of the Tissue of Origin Endometrial Test An ROC curve (A) for the Tissue of Origin Endometrial Test using results from the independent validation specimens gives an AUC value of 0.997. Distribution of similarity scores (B) obtained for the validation specimens shows clear separation of ovarian and endometrial cancer predictions.

An ROC for the Tissue of Origin Endometrial Test was plotted and an AUC of 0.997 was obtained indicating high discriminatory performance between ovarian and endometrial cancers (Figure 3A). The DOR of 406 also indicated that the Tissue of Origin Endometrial Test had good discrimination between ovarian and endometrial cancers.

A distribution plot of the endometrial similarity scores in bins of 10 for the 75 clinical validation specimens is plotted in Figure 3B. Since the sum of the similarity scores for endometrial and ovarian tissue types is 100, the higher the endometrial similarity score the lower the ovarian similarity score. The median higher similarity score for cases that gave agreements with the known clinical diagnosis is 95.6 (range – 70.4-99.3) while the median higher similarity score for cases that gave non-agreements was much lower at 60.8 (range – 59.6-71.6). Eighty nine percent (67/75) of the specimens in the validation set had a higher similarity score that was greater than 80. Among the ovarian predictions, 25/30 specimens had similarity scores of >90 while 31/45 specimens that gave endometrial predictions had similarity scores of >90. A clear separation in the higher similarity score was observed for the vast majority of ovarian and endometrial cancer cases (Figure 3B). When the higher similarity score is greater than 80, the probability of agreement between the Tissue of Origin Endometrial Test result and the known clinical diagnosis was 100%. This also indicates that a similarity score of ≤ 20 rules out that tissue type with > 99% probability (95% CI, 94.6% to 100%).

### Performance of the Tissue of Origin Endometrial Test According to Specimen Attributes

The majority of the specimens used for clinical validation were acquired in the prior 2 years (Table 4). For specimens >2 years old, Tissue of Origin Endometrial Test performance was 90.5%. This group included two specimens that were 7 years old and both of these specimens gave accurate Tissue of Origin Endometrial Test results. The age of the specimen did not affect Test performance.

**Table 4:** Tissue of Origin Endometrial Test Performance According to Specimen Attributes

Specimen Attribute	Category Definition	N	PPA Percent, (Ratio) [95% Confidence Interval]	Percent Non-Agreement Percent, (Ratio) [95% Confidence Interval]
**Age of Specimen**	<1 year	24	95.8 (23/24) [78.9-99.9]	4.2 (1/24) [0.1-21.1]
	1-2 year	30	96.7 (29/30) [82.8-99.9]	3.3 (1/30) [0.1-17.2]
	>2 year	21	90.5 (19/21) [69.6-98.8]	9.5 (2/21) [1.2-30.4]
**Percent Tumor Content**	90% < × ≤ 100%	13	92.3 (12/13) [64.0-99.8]	7.7 (1/13) [0.2-36.0]
	80% < × ≤ 90%	22	95.5 (21/22) [77.2-99.9]	4.5 (1/22) [0.1-22.8]
	70% < × ≤ 80%	21	90.5 (19/21) [69.6-98.8]	9.5 (2/21) [1.2-30.4]
	60% ≤ × ≤ 70%	19	100 (19/19) [85.4-100]	0.0 (0/19) [0.0-14.6]
**Percent Necrosis Content**	0% ≤ × < 10%	48	93.8 (45/48) [82.8-98.7]	6.3 (3/48) [1.3-17.2]
	10% ≤ × < 20%	18	94.4 (17/18) [72.7- 99.9]	5.6 (1/18) [0.1-27.3]
	20% ≤ × < 30%	4	100 (4/4) [39.8-100]	0.0 (0/4) [0.0-60.2]
	30% ≤ × ≤ 40%	5	100 (5/5) [47.8-100]	0.0 (0/5) [0.0-52.2]

PPA, Positive percent agreement; N, Number of specimens in each category

Test performance was also consistent at all levels of viable percent tumor above the minimal threshold (≥ 60%) of tumor content required for specimen entry into the Tissue of Origin Endometrial Test (Table 4). Specimens in the 60% to 70% tumor content bin had a PPA of 100%. The presence of up to 40% necrosis in the specimen under analysis did not diminish performance of the Tissue of Origin Endometrial Test (Table 4).

### Performance of the Tissue of Origin Endometrial Test According to Patient and Cancer Attributes

**Figure 4:**
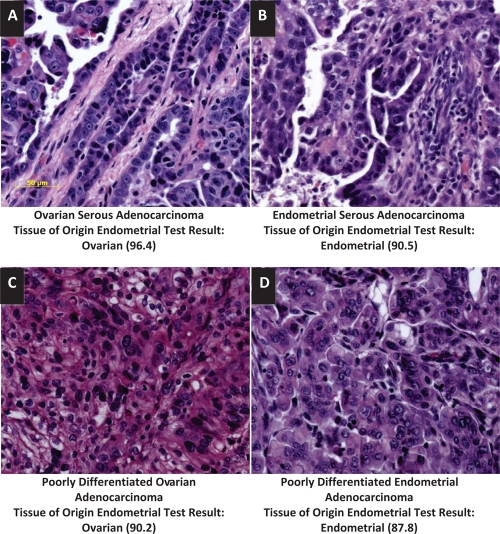
Distinction of ovarian and endometrial cancers by the Tissue of Origin Endometrial Test H&E stained sections (A, B, C and D) of an ovarian serous adenocarcinoma (A), an endometrial serous adenocarcinoma (B), a poorly differentiated ovarian adenocarcinoma (C) and a poorly differentiated endometrial adenocarcinoma (D) from the validation specimens show similar morphologic appearance of ovarian and endometrial serous adenocarcinomas (A and B) and ovarian and endometrial poorly differentiated adenocarcinomas (C and D). The Tissue of Origin Endometrial Test clearly distinguished between these tumors. The Tissue of Origin Endometrial Test result was ovarian with a similarity score of 96.4 for the ovarian serous adenocarcinoma (A), ovarian with a similarity score of 90.2 for the poorly differentiated ovarian adenocarcinoms (C), endometrial with a similarity score of 90.5 for the endometrial serous adenocarcinoma (B) and endometrial with a similarity score of 87.8 for the poorly differentiated endometrial adenocarcinoma (D). All images are at the same magnification. Bar = 50 μm.

The age range of patients from whom validation specimens were obtained was wide with the majority of the patients being 50 to 80 years old (Table 5). The Tissue of Origin Endometrial Test had good performance for all patient age groups (Table 5). The grade of the cancer was known for 70/75 cases included in the validation study. Since primary tumors in the validation set were solely composed of poorly differentiated to undifferentiated cancers, the vast majority of cases were Grade 3 cancers. The Tissue of Origin Endometrial Test had high accuracy for both Grade 3 ovarian and Grade 3 endometrial cancers (Table 5). Tissue of Origin Endometrial Test performance was good with both metastatic specimens (90.5%; n=21) and with poorly differentiated and undifferentiated primary tumors (96.3%; n=54) (Table 5). There were a larger number of metastatic ovarian tumors compared to metastatic endometrial tumors (Table 5). The difference in PPA between metastatic specimens of ovarian (94.1%; n=17) and endometrial (75%; n=4) origins is not statistically significant (Fisher's exact test, p=0.3524). The majority of metastatic samples (13/21) were biopsied from the omentum and these samples had a 100% (95% CI, 75.3-100) agreement with the known clinical diagnosis. Other metastatic specimens were either lung (n=4) or soft tissue (n=4) biopsies. Both of these biopsy sites gave three (of 4) agreements each with the available clinical diagnosis. The stage of the cancer was known for 50/75 cases included in the validation set. The majority of ovarian cases used in the validation study were Stage III and IV cancers (n=21) while the majority of endometrial cases were Stage I cancers (n=15). However, the validation set included cases that were representative of all stages of ovarian and endometrial cancers and the Tissue of Origin Endometrial Test had good performance with all stages of ovarian and endometrial cancers. The validation set included both ovarian serous adenocarcinomas (n=22) and endometrial serous adenocarcinomas (n=7). The Tissue of Origin Endometrial Test clearly distinguished serous adenocarcinomas of ovarian and endometrial origins even when a clear distinction could not be made based on histologic criteria (Table 5, Figure 4). The difference in PPA between ovarian serous adenocarcinomas (100%) and endometrial serous adenocarcinomas (71.4%) was not statistically significant (Fisher's exact test, p=0.0517). The Tissue of Origin Endometrial Test could also distinguish between poorly differentiated ovarian and poorly differentiated endometrial cancers (Figure 4).

**Table 5:** Tissue of Origin Endometrial Test Performance According to Patient and Cancer Attributes

Patient/Cancer Attribute	Category Definition	N	Ovarian PPA Percent, (Ratio) [95% Confidence Interval]	Endometrial PPA Percent, (Ratio) [95% Confidence Interval]	Overall PPA Percent, (Ratio) [95% Confidence Interval]	p-value*
**Age of Patient (years)**	30-50	9	100 (5/5) [47.8-100]	100 (4/4) [39.8-100]	100 (9/9) [66.4-100]	
	50-60	24	100 (11/11) [71.5-100]	100 (13/13) [75.3-100]	100 (24/24) [85.8-100]	
	60-70	28	100 (11/11) [63.6-98.5]	88.2 (15/17) [63.6-98.5]	92.9 (26/28) [76.5-99.1]	0.505
	70-90	14	75 (2/3) [9.4-99.2]	90.9 (10/11) [58.7-99.8]	85.7 (12/14) [57.2-98.2]	0.396
**Cancer Grade**	Grade 1	0				
	Grade 2	2	100 (2/2) [15.8-100]	100 (1/1) [2.5-100]	100 (3/3) [29.2-100]	
	Grade 3	25	96 (24/25) [79.6-99.9]	95.2 (40/42) [83.8-99.4]	95.5 (64/67) [87.5-99.1]	1.000
**Primary versus Metastatic Cancer**	Metastatic	21	94.1 (16/17) [71.3-99.9]	75.0 (3/4) [19.4-99.4]	90.5 (19/21) [69.6-98.8]	0.352
	Primary	54	100 (13/13) [75.3-100]	95.1 (39/41) [83.5-99.4]	96.3 (52/54) [87.3-99.5]	1.000
**Cancer Stage**	I	19	100 (4/4) [39.8-100]	100 (15/15) [78.2-100]	100 (19/19) [82.4-100]	
	II	7	100 (3/3) [29.2-100]	75.0 (3/4) [19.4-99.4]	85.7 (6/7) [42.1-99.6]	1.000
	III and IV	24	95.2 (20/21) [76.2-99.9]	66.7 (2/3) [9.4-99.2]	91.7 (22/24) [73.0-99.0]	0.239
**Cancer Morphology**	Serous	29	100 (22/22) [84.6-100]	71.4 (5/7) [29.0-96.3]	93.1 (27/29) [77.2-99.2]	0.052
	Other	46	87.5 (7/8) [47.3-99.7]	97.4 (37/38) [86.2-99.9]	95.7 (44/46) [85.2-99.5]	0.321

PPA, positive percent agreement; N, Number of Specimens in each category

* A fisher's exact t-test was used to calculate a p-value for the difference in PPA between ovarian and endometrial cancers

### Reproducibility of the Tissue of Origin Endometrial Test

Intra-site reproducibility (precision) was assessed by performing pairwise comparisons of the Tissue of Origin Endometrial Test results obtained from 3 adjacent sections from the same tissue block processed in the same run. Percent concordance in test results was 100% for each pairwise comparison (Table 6). The median coefficient of variation (CV%) of the similarity score for the known clinical diagnosis was 1.38 (range 0.32 to 8.79). The value of Kappa (Κ) statistics was 1.0 for all three pairwise comparisons indicating perfect agreement in the Tissue of Origin Endometrial Test results between sections processed in the same run.

**Table 6:** Reproducibility of the Tissue of Origin Endometrial Test

Reproducibility of the Tissue of Origin Endometrial Test			
**Study**	Comparison	Concordance Percent (Ratio) [95% Confidence Interval]	Discordance Percent (Ratio) [95% Confidence Interval]
**Intra-Site Reproducibility**	Section # 2 versus Section # 3	100 (15/15) [78.2-100.0]	0 (0/15) [0.0-21.8]
	Section # 2 versus Section # 4	100 (16/16) [79.4-100.0]	0 (0/16) [0.0-20.6]
	Section # 3 versus Section # 4	100 (15/15) [78.2-100.0]	0 (0/15) [0.0-21.8]
	Overall	100 (46/46) [92.3-100.0]	0 (0/46) [0.0-7.7]
**Inter-Site Reproducibility**	PWDL versus EA	93.3 (28/30) [77.9-99.2]	6.6 (2/30) [0.8-22.1]
	PWDL versus GLGC	96.6 (28/29) [82.2-99.9]	3.4 (1/29) [0.1-17.8]
	EA versus GLGC	93.1 (27/29) [77.2-99.2]	6.9 (2/29) [0.8-22.8]
	Overall	94.3 (83/88) [87.2-98.1]	5.7 (5/88) [1.9-12.8]

PWDL, Pathwork Diagnostics Laboratory; EA, Expression Analysis; GLGC, GeneLogic

Pairwise comparisons of Tissue of Origin Endometrial test results for adjacent sections from 30 specimens processed at three laboratories (PWDL, EA and GLGC) were performed to assess inter-site reproducibility. The percent concordance in test results for PWDL versus EA was 93.3%, for PWDL versus GLGC was 96.6% and for EA versus GLGC was 93.1% (Table 6). The median CV% of the similarity score for the known clinical diagnosis was 3.02 (range – 0.23 to 46.9%). Twenty seven specimens had Tissue of Origin Endometrial Test results that were concordant at all 3 sites, and all 27 specimens had CV% that were less than 10. Kappa (Κ) statistics for agreement for PWDL versus EA was 0.87 (95% CI, 0.66-1.00), for PWDL versus GLGC was 0.93 (95% CI, 0.76-1.00) and for EA versus GLGC was 0.86 (95% CI, 0.65-1.00), indicating a very good agreement (Κ ≥ 0.81) in Tissue of Origin Endometrial Test results between the three sites.

## DISCUSSION

The Pathwork Tissue of Origin Endometrial Test is the first microarray-based diagnostic test that has been successfully used to differentiate ovarian from endometrial cancers. Performance was evaluated in an independent set of specimens that were solely composed of either metastatic cancers or poorly differentiated and undifferentiated primary cancers [10]. The Tissue of Origin Endometrial Test showed a high degree of agreement with the clinically available diagnosis for these challenging specimens, accurately identifying the primary site for 94.7% of ovarian and endometrial cancers. In addition to the high accuracy, there was a clear separation in similarity scores obtained for the 2 tissue types.

The Tissue of Origin Endometrial Test uses 375 probesets (316 independent genes) to classify ovarian and endometrial cancers. These classification biomarkers were chosen based upon maximal performance observed with the training data. The classification biomarkers were empirically selected. Nonetheless, they included several genes with known functional roles in ovarian and/or endometrial cancers as well as the common immunohistochemical biomarker, WT1, that is used to distinguish ovarian serous and endometrial serous adenocarcinomas [6,29,31].

The Tissue of Origin Endometrial Test utilizes FFPE specimens, the most commonly available clinical specimen. Additionally, the success rate for processing of FFPE specimens that had met specimen entry criteria was 96.3%. The use of FFPE along with a high processing success rate is advantageous in the clinical setting, and will allow for wide usage of the Tissue of Origin Endometrial Test.

The Tissue of Origin Endometrial Test includes 14 different WHO histologic subtypes of ovarian and endometrial cancers. Poorly differentiated adenocarcinomas of ovarian and endometrial origins can be difficult to distinguish because of similar histologic appearances. In addition, endometrioid ovarian and endometrioid endometrial subtypes as well as serous ovarian and serous endometrial subtypes are histologically similar and their primary sites can be difficult to diagnose [3,4,6]. The Tissue of Origin Endometrial Test training and test sets included both ovarian and endometrial serous histologic subtypes and also both ovarian and endometrial endometrioid histologic subtypes. Test set analysis showed that the Tissue of Origin Endometrial Test can distinguish between ovarian and endometrial endometrioid cancers and also between ovarian and endometrial serous cancers (data not shown). The Tissue of Origin Endometrial Test validation set did not include any ovarian endometrioid carcinomas but included both ovarian serous (N=22) and endometrial serous (N=7) carcinomas. The test identified all ovarian serous cancers and 5 endometrial serous cancers accurately. Thus, the Tissue of Origin Endometrial test can distinguish serous morphologies from ovarian and endometrial origins. It also performed well with poorly differentiated cancers of ovarian and endometrial origins.

In this study, we present data validating the accuracy and reproducibility of the Tissue of Origin Endometrial Test. Clinical management and treatment choices for ovarian and endometrial cancer patients are different with endometrial cancer patients often receiving radiation therapy and possibly hormonal therapy. Future studies and clinical use of the Tissue of Origin Endometrial Test will ultimately determine how this test impacts clinical management of ovarian and endometrial cancer patients. We anticipate the Tissue of Origin Endometrial Test to have great clinical value for patients whose cancers involve both the ovary and uterus. In these cases, the test would distinguish between simultaneous primary malignancies or metastatic spread from either the ovary or the endometrium. As more targeted therapies specific for ovarian or endometrial cancers are developed, the Tissue of Origin Endometrial Test is expected to have increasing impact on the clinical outcome and life expectancy of these patients.

## METHODS

### FFPE Tumor Specimens

FFPE tumor specimens, acquired using Institutional Review Board-approved procedures, were obtained from six different human tumor tissue banks. All specimens were excisional biopsies, had a known ovarian or endometrial cancer clinical diagnosis and were from one of the included morphologies (see Table 1) on the Tissue of Origin Endometrial Test panel. All specimens had a known biopsy site and were either metastatic tumors or primary tumors that were poorly differentiated or undifferentiated. Available clinical information associated with the specimen including age, race, tissue dimensions and date (year) of resection was recorded. H&E sections adjacent to the tumor sample were reviewed by a pathologist to determine the percentage of tumor tissue, normal tissue and necrosis. Specimen requirement for entry into the Tissue of Origin Endometrial Test includes a minimum of 60% tumor tissue. Therefore, only specimens that contained at least 60% tumor tissue were used in this study. The set of specimens used for algorithm training was completely independent from the set of specimens used for clinical validation.

### RNA Extraction, Target Preparation, and Microarray Processing Procedures

FFPE tumor specimens were processed as described in a previous study [16]. Briefly, total RNA was isolated from 10 μm thick sections using the Agencourt FormaPure system (Beckman Coulter Genomics, Beverly, MA) and the Ambion DNase I RNA-free kit (Life Technologies, Austin, TX). Total RNA concentration was assessed by spectrophotometry (OD 260 nm, NanoDrop, Wilmington, DE), and the purity was judged by the ratio of absorbance at 260 nm to 280 nm (A_260_/A_280_). A minimum total RNA yield of 30 ng at a concentration of ≥ 9.5 ng/μl and with an A_260_/A_280_ ratio of ≥ 1.0 was required to proceed to target preparation. Thirty ng of total RNA was amplified using the RampUp kit (Genisphere, Hatfield, PA) to generate biotin-labeled cDNA. A minimum yield of 2.5 μg of labeled cDNA was required to proceed to microarray analysis. Labeled cDNA was hybridized to a Pathchip® microarray (manufactured by Affymetrix, Santa Clara, CA), and washed and stained using commercially available reagent kits and protocols (Affymetrix, Inc., Santa Clara, CA). The arrays were scanned using either the Affymetrix GCS3000Dx or the Affymetrix 7G Scanner. The resulting raw intensity data files (CEL files) were analyzed for data quality. Data quality was verified to meet prespecified quality control metrics of Overall Signal ≥ 10, Percent Present ≥ 5, and Regional Discontinuity ≤ 0.84, calculated as described previously [16].

### Specimen Processing Sites

All specimen preparation and processing was performed at one of three independent processing laboratories: Pathwork Diagnostics Laboratory (PWDL, Redwood City, CA), Expression Analysis (EA, Durham, NC) and GeneLogic, Inc (GLGC, Gaithersburg, MA). PWDL and GLGC processed specimens for the clinical validation study; inter-site reproducibility was assessed at PWDL, EA and GLGC; intra-site repeatability (precision) was assessed at PWDL. Laboratories performing the test were blinded to the known clinical diagnosis. All CEL files were transferred to Pathwork Diagnostics for data quality assessment and analysis through the Tissue of Origin Endometrial Test.

### Tissue of Origin Endometrial Test Algorithm Development

The Pathwork Tissue of Origin Endometrial Test indicates whether Ovary or Endometrium is the more likely tissue of origin. The test relies on two distinct algorithms, one for standardization and one for classification. The standardization algorithm utilizes a set of 61 stable probe-sets to normalize the raw probe-level intensity values of the gene expression profiles under analysis and reduce technical variation incurred by different processing conditions. The standardization algorithm was developed by evaluating >5000 tissue specimens from a range of tissue types that were processed at 11 laboratories. The standardization expression values for each probe-set generated by the standardization algorithm are used by the Tissue of Origin Endometrial Test classification algorithm. The classification algorithm was developed using a database of 484 ovarian and 365 endometrial specimens that had a known ovarian or endometrial cancer diagnosis based on clinical history and were from one of the included morphologies on the Tissue of Origin Endometrial Test panel (Table 1). A machine learning approach was used to select the optimal model for classifying ovarian and endometrial cancers. The optimal model consisted of a list of 375 probe-sets (316 independent genes) and a set of coefficients that are combined to produce 2 similarity scores. The 2 similarity scores correspond to the probability that the gene expression profile of the input specimen matches the expression profile of ovarian and endometrial cancers [16]. The standardization and classification algorithms were locked prior to initiation of clinical validation studies.

### Tissue of Origin Endometrial Test Report

The Tissue of Origin Endometrial Test report (Figure 1) is interpreted using the following guide to report interpretation: The Similarity Score (SS) is a measure of the similarity of the RNA expression pattern of the specimen to the RNA expression patterns of endometrial and ovarian tumor tissues. Two SS are generated, one for endometrial origin and one for ovarian origin. The scores add up to 100. The higher the SS, the more likely it represents the tissue of origin of the cancer.

### Clinical Validation Study Design

The Tissue of Origin Endometrial Test clinical validation study used Bayesian adaptive analysis to determine the design plan and sample sizes [33,34]. Details regarding the clinical validation study design and sample size estimates are provided in Supplementary Methods and Supplementary Table 1. Briefly, the study was designed to have no greater than 5% Type I error and at least 98% power to meet prespecified acceptance criteria. The criteria are based on the mean positive percent agreement (mean PPA) between the Test prediction and the available clinical diagnosis. The acceptance criteria were: at least 80% mean PPA and at least 65% lower bound of the corresponding 95% credible interval. These requirements were derived from analyses of the training data, requirements for clinical utility of the test, and practicality of obtaining sufficient number of specimens. The study design used three phases, commonly referred to as Looks. Look 1 uses ½ of the maximum sample size; Look 2 uses ¾ of the maximum sample size; and Look 3 uses the maximum sample size. The study design allowed for early termination based on intermediate analyses after each look. Study termination at each look could occur if the acceptance criteria were met or if the predicted probability of success at the maximum sample size, based on the data accumulated up to that point, was less than 5%.

### Reproducibility Study Design

The intra-site (precision) and inter-site reproducibility of the Tissue of Origin Endometrial Test was assessed as follows: For intra-site reproducibility (precision), three adjacent 10 μm thick sections from FFPE tissue blocks of 16 specimens (ovarian=8; endometrial=8) that gave an agreement with the known clinical diagnosis were processed simultaneously within the same run. For inter-site reproducibility, three adjacent 10 μm thick sections from FFPE tissue blocks of 30 specimens (ovarian=15; endometrial=15) that gave an agreement with the known clinical diagnosis were processed at each of the three laboratories in this study. Concordance in Tissue of Origin Endometrial Test results obtained from pairwise comparisons of the three sections was used as a measure of test reproducibility for both the intra-site and inter-site analysis. Details regarding sample size estimates for the reproducibility study are provided in Supplementary Methods.

## DATA ANALYSIS

Mean PPA and credible intervals are Bayesian parameters that were used as the primary endpoint for the Tissue of Origin Endometrial Test clinical validation study (see Supplementary Methods for details). Performance of the Test was measured as the positive percent agreement (PPA) which is defined as the percent agreement between the Tissue of Origin Endometrial Test result and the known clinical diagnosis. A receiver operator curve (ROC) was plotted and AUC was calculated using SigmaPlot 12 [35]. The DOR was calculated to provide a single indicator of test performance as described earlier [36]. For intra-site and inter-site reproducibility, results were considered concordant if the Tissue of Origin Endometrial test results from one section or site matched the result from another section or site. In both cases, an overall pairwise percent concordance in Tissue of Origin Endometrial Test results is reported. Kappa statistics for agreement were calculated for pairwise comparisons using R Version 2.11.1 (2010-05-31) [37].

## Supplementary Tables




