# Supplementary material for: A Gene Expression Profile Test for the Differential Diagnosis of Ovarian Versus Endometrial Cancers
Source: Oncotarget. 2012 Feb 23;3(2):212–23. doi: 10.18632/oncotarget.450 (PMC3326651; doi:10.18632/oncotarget.450)
Supplement: Supplementary file 1 [file oncotarget-03-212-s001.docx]

| **Supplementary Table 1:** Sample Size Estimates and Bayesian Adaptive Design for Clinical Validation of the Tissue of Origin Endometrial Test | | | | | |
| --- | --- | --- | --- | --- | --- |
| **Look** | **Sample Size** | **FutilityBound*** | **Pr (Lose)**** | **Pr (Win)***** | **Pr(Type I Error)^$^** |
|  | | | | | |
| **OVARIAN CANCER** | | | | | |
| 1 | 30 | 21 | 0.001 | 0.94 | 0.023 |
| 2 | 45 | 34 | 0.002 | 0.983 | 0.027 |
| 3 | 59 | 47 | 0.003 | 0.994 | 0.028 |
|  | | | | | |
| **ENDOMETRIAL CANCER** | | | | | |
| 1 | 45 | 33 | 0.004 | 0.935 | 0.009 |
| 2 | 67 | 51 | 0.002 | 0.974 | 0.01 |
| 3 | 89 | 71 | 0.004 | 0.991 | 0.01 |
| * FutilityBound is the minimum number of agreements with the available clinical diagnosis needed to proceed to the next Look. **Pr (Lose) is the cumulative probability that the number of agreements will be less than or equal to the FutilityBound. ***Pr (Win) is the cumulative probability that the acceptance criteria will be met by sample size N. ^$^Pr (Type I Error) is cumulative probability of Type I error for the sample size N. | | | | | |
