# Supplementary material for: A Gene Expression Profile Test for the Differential Diagnosis of Ovarian Versus Endometrial Cancers
Source: Oncotarget. 2012 Feb 23;3(2):212–23. doi: 10.18632/oncotarget.450 (PMC3326651; doi:10.18632/oncotarget.450)
Supplement: Supplementary file 2 [file oncotarget-03-212-s002.docx]

| **Supplementary Table 2:** Bayesian Performance Characteristics for the Clinical Validation of the Tissue of Origin Endometrial Test | | |
| --- | --- | --- |
| **Known Clinical Diagnosis** | **Mean PPA** | **Mean Percent Non-Agreement Percent (ratio)** |
|  | **Percent (ratio)** | **[95% Credible Interval]** |
|  | **[95% Credible Interval]** |  |
| Endometrial | 91.5 (42/45) | 8.5 (3/45) |
|  | [82.1-97.6] | [2.4-17.9] |
| Ovarian | 93.8 (29/30) | 6.2 (1/30) |
|  | [83.3-99.2] | [0.8-16.7] |
| Overall | 93.5 (71/75) | 6.5 (4/75) |
|  | [87.1-97.8] | [2.2-12.9] |
|  |  |  |
|  |  |  |
| Lal et al. A Gene Expression Profile Test for the Differential Diagnosis of Ovarian Versus Endometrial Cancers | | |
